# Modelling the trends of inpatient and outpatient rehabilitation for methamphetamine in the Western Cape province of South Africa

**DOI:** 10.1186/s13104-015-1741-4

**Published:** 2015-12-18

**Authors:** J. Mushanyu, F. Nyabadza, A. G. R. Stewart

**Affiliations:** Department of Mathematics, University of Zimbabwe, Box MP 167, Mount Pleasant, Harare, Zimbabwe; Department of Mathematical Sciences, Stellenbosch University, P. Bag X1, Matieland, 7602 South Africa

**Keywords:** Methamphetamine, Reproduction number, Inpatient rehabilitation, Outpatient rehabilitation, Least squares curve fitting

## Abstract

**Background:**

Dependence on methamphetamine remains one of the major health and social problem in the Western Cape province of South Africa. We consider a mathematical model that takes into account two forms of rehabilitation, namely; inpatient and outpatient. We examine the trends of these two types of rehabilitation. We also seek to investigate the global dynamics of the developed methamphetamine epidemic model.

**Methods:**

The model is designed by likening the initiation process to an infection that spreads in a community through interactions between methamphetamine users and non-users. We make use of Lyapunov functions obtained from a suitable combination of common quadratic and Volterra-type functions to establish the global stability of the methamphetamine-persistent steady state. The least squares curve fit routine (lsqcurvefit) in Matlab with optimization is used to estimate the parameter values.

**Results:**

The model analysis shows that the model has two equilibria, the methamphetamine free equilibrium and the methamphetamine persistent equilibrium, that are both globally stable when the threshold $\mathcal {R}_a<1$ and $\mathcal {R}_a>1$, respectively. Upon fitting the model to data on drug users under rehabilitation, parameter values that give the best fit were obtained. The projections carried out the long term trends of these forms of rehabilitation.

**Conclusion:**

The results suggest that inpatient rehabilitation programs have an increased potential of enhancing the chances of recovery for methamphetamine addicts.

## Background

The methamphetamine abuse problem and its consequences to communities in the Western Cape province present a complex scenario that drives sexually transmitted infections morbidity, mortality and heavy budgetary constraints. For example in South Africa over R20 billion is used annually, in treating drug users, cracking down drug traffickers and in prevention and media campaigns [1]. In the Western Cape Province of South Africa, the rising demand for substance abuse services and calls by communities for additional services has led to the Provincial Department of Social Development allocating additional resources to the prevention and treatment of substance use disorders (SUD’s). However, planning around the allocation of these resources has been hampered by several informational barriers. Decision makers within the Western Cape Department of Social Development (DOSD) do not have adequate information on the nature and extent of substance abuse in the province, the extent to which there is unmet substance abuse service needs, where these unmet needs are greatest, and which population subgroups have relatively high unmet needs [2]. There is therefore need for extensive research into methamphetamine abuse trends for the development of public policies and focused correctional services to decrease the gang populations.

Recently, many researchers have drawn a lot of scholarship from infectious diseases modelling with the aim of describing drug abuse spreading like an infectious disease [3–5]. Typical examples are models designed for heroin epidemics [12–15], peer influence [6–9] and the spread of alcoholism [10, 11]. Mathematical models can contribute to the understanding of the various aspects of methamphetamine use and are very useful in determining how prevalent drug use is. They can even help in designing and choosing proper interventions by providing a means of integrating data from different sources, describing a process to increase understanding and simulating policy experiments that are cumbersome in real life [16–18].

**Fig. 1:**
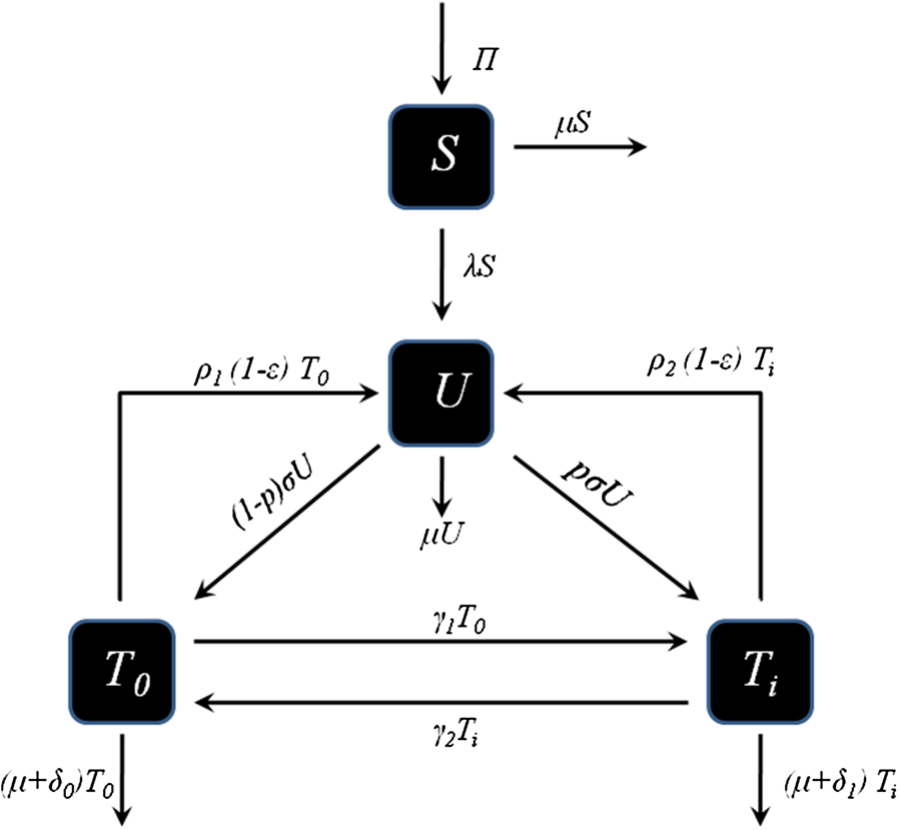
Model flow diagram. Schematic diagram showing the movement of humans as their status with respect to drug use changes

**Fig. 2:**
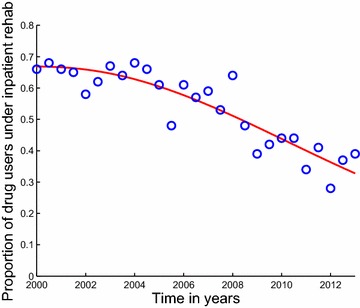
Model system Eqs. (2)–(5) fitted to data for individuals under inpatient rehabilitation in Cape Town. The *blue circles* indicate the actual data and the *solid red line* indicates the model fit to the data. The percentages are not of all users, but of those in rehabilitation. From the second half (July–December) of the year 2008 back to the first half (January–June) of the year 2000, more than half of the patients admitted in the treatment centers of Cape Town were treated on an inpatient basis. It shows a decline in the proportion of drug users treated under inpatient rehabilitation programs in Cape Town, see also Fig. 6. This decline, might be a result of retarding economic situation or expensive costs associated with inpatient rehabilitation centers. Also, due to the fact that inpatient rehabilitation programs are formally 24 h programs, some people would prefer outpatient rehabilitation programs to allow them to work for their families whilst receiving treatment. It demonstrates a good fit to the data from Table 1

There are two primary forms of rehabilitation for methamphetamine addiction namely; inpatient and outpatient rehabilitation [19]. Rehabilitation helps individuals to improve their function, mobility, independence and quality of life. It helps individuals live fully regardless of impairment. It helps people who are living with various health conditions to maintain the functioning they have [20]. Inpatient methamphetamine rehabilitation provides an addict a place to live and at the same time providing 24 h support and treatment. Outpatient methamphetamine rehabilitation allows users to reside at home and come to the treatment center on a regular basis [19] (Figs. 1, 2).

Mathematical models on drug abuse that have been developed so far, have unified rehabilitation, see for instance [14, 15, 21, 31]. In this paper, we develop a mathematical model that takes into account both inpatient and outpatient forms of rehabilitation. Our aim is to examine the trends of these two types of rehabilitation. We also seek to investigate the global dynamics of the developed methamphetamine epidemic model. We analyze the global stability of the model equilibria and fit the model to data on these two forms of rehabilitation.

**Fig. 3:**
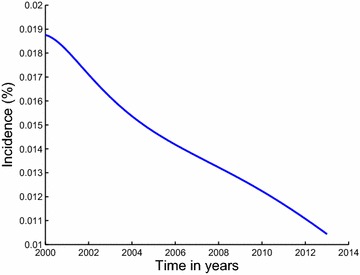
Estimated incidence of methamphetamine abuse using data for inpatient rehabilitants in Cape Town. Our estimated incidence of methamphetamine abuse, evaluated using the initiation function $\lambda S$, is observed to be generally decreasing over the modeling period

**Fig. 4:**
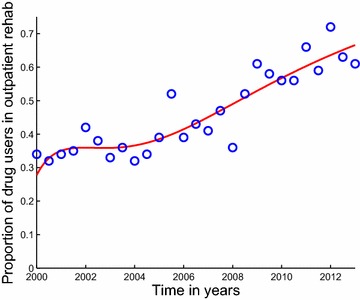
Model system Eqs. (2)–(5) fitted to data for individuals under outpatient rehabilitation in Cape Town. The *blue circles* indicate the actual data and the *solid red line* indicates the model fit to the data. The percentages are not of all users, but of those in rehabilitation. Starting from the second half (July–December) of the year 2008 up to the first half (January–June) of the year 2013, the majority of patients in specialist treatment centres of Cape Town were now being treated on an outpatient basis. It shows a good fit to the data from Table 1

The paper is arranged as follows; in Sect. “Methods”, we formulate and establish the basic properties of the model. The model is analysed for stability in Sect. “Model analysis”. Parameter estimation and projection graphs are given in Sect. “Results and discussion”. Numerical results are also presented in this section. The paper is concluded in Sect. “Conclusions” (Figs. 3, 4).

## Methods

### Model formulation

The dynamics of inpatient and outpatient rehabilitation are modelled by considering the human population divided into four distinct compartments. The compartments comprise of *S* denoting the population at risk of being initiated into methamphetamine abuse, *U* those initiated into methamphetamine abuse, $T_0$ those in rehabilitation as out-patients and $T_i$ those in rehabilitation as in-patients. Here, all susceptible individuals range from age 15–64 years. The total human population is thus given by

$$\begin{aligned} N=S+U+T_0+T_i. \end{aligned}$$

We assume that individuals in each compartment are indistinguishable and there is homogeneous mixing. Susceptible humans enter the population through births or immigration at a rate $\Pi$. Susceptible individuals are initiated into methamphetamine use following interaction with individuals using drugs. We assume an initiation function that is analogous to the force of infection for epidemic models. Thus, the per capita contact rate $\beta$ is a product of the effective number of contacts *c*, between methamphetamine users and the susceptible population, and the probability $\hat{\beta }$, that a contact results into initiation into methamphetamine use, that is, $\beta =c\hat{\beta }$. A fraction *U*/*N* of the contacts is with those drug users not in rehabilitation and the average number of potential contacts resulting in susceptible individuals becoming methamphetamine abusers is $\beta U/N$. Also, a fraction $T_0/N$ of the contacts is with individuals under outpatient rehabilitation. The average number of potential contacts of each susceptible individual with individuals in outpatient rehabilitation is $\beta \eta T_0/N$. The parameter $\eta$ measures the relative ability for individuals in outpatient rehabilitation to initiate new methamphetamine users. Assuming that the rate at which individuals in outpatient rehabilitation recruit initiates is lower than that for drug users not in rehabilitation, we have, $0<\eta <1$. This is due to the fact that individuals in outpatient rehabilitation will be receiving some form of counselling and therapy to aid them in quitting drug abuse. Such counselling may lead them to discourage susceptible individuals into becoming methamphetamine abusers. This done by raising awareness on the dangers associated with problematic drug use. Here, we make an assumption that individuals under inpatient rehabilitation cannot produce new initiates due to the fact that, they do not come in contact with the general population during the rehabilitation process. The total number of relevant contacts gives the initiation function,

1 $$\begin{aligned} \lambda \,=\,\beta \left( \dfrac{U+\eta T_0}{N}\right) . \end{aligned}$$

Upon being initiated into methamphetamine use, a susceptible individual moves into the compartment *U*, of methamphetamine abusers. Upon realizing the demoralizing consequences related to methamphetamine abuse, individuals in the compartment *U* seek help via rehabilitation programs. We consider two primary types of rehabilitation programs, namely; inpatient and outpatient forms of rehabilitation. Here, we define the effectiveness of these rehabilitation programs to be, “the measure of the benefits and changes in the functioning of an individual acquired during the period when he/she was under treatment”. The effectiveness of rehabilitation programs is measured by the parameter $\varepsilon$. If $\varepsilon = 0$, then the rehabilitation programs are not effective, $\varepsilon = 1$ corresponds to completely effective rehabilitation programs, while $0<\varepsilon <1$ implies that rehabilitation programs will be effective to some degree. Amongst individuals in compartment *U* who are seeking help through rehabilitation, we have that a proportion *p* of these individuals are recruited into inpatient rehabilitation facilities and the complementary proportion $(1-p)$ are recruited into outpatient rehabilitation. The rate at which methamphetamine users are recruited into rehabilitation (inpatient or outpatient) is given by $\sigma$. Once an individual is in the rehabilitation phase, he/she can either quit permanently, relapse into methamphetamine use or die. Individuals experience natural death at a rate $\mu$. The rate at which individuals under outpatient rehabilitation quit methamphetamine abuse permanently is represented by $\delta _0$ and the rate at which those under inpatient rehabilitation quit methamphetamine abuse permanently is represented by $\delta _1$. Individuals undergoing outpatient rehabilitation relapse into methamphetamine use at a rate $\rho _1$ and individuals in inpatient rehabilitation facilities relapse into methamphetamine use at a rate $\rho _2$. Substance abusers undergoing outpatient rehabilitation continue to keep contact with people and circumstances that trigger addiction whereas substance abusers undergoing inpatient rehabilitation are completely immersed in the program and separated from the lifestyle and habits that supported drug use, thus, relapsing may be more likely for outpatient rehabilitants as compared to inpatient rehabilitants. We can safely assume that $\rho _2<\rho _1$. Individuals under inpatient rehabilitation programs can move to outpatient rehabilitation programs at a rate $\gamma _2$. This movement may be as a result of some individuals failing to cope up with the higher costs usually associated with inpatient rehabilitation and thereby forcing them rather to seek help through outpatient rehabilitation which is generally cheaper. This movement might also be as a result of noted recovery to some individuals, who if discharged can still fully recover whilst residing at home. The rate at which individuals under outpatient rehabilitation move to inpatient rehabilitation facilities is given by $\gamma _1$. This movement can be due to the fact that some individuals under outpatient rehabilitation might want to take full advantage of all the resources available in treatment, such as personal therapy, group therapy, educational classes on addiction as well as job skills and other related support structures in order to quicken their recovery process. So this in turn will lead them to seek help from inpatient rehabilitation facilities.

**Fig. 5:**
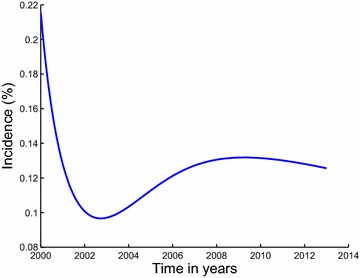
Estimated incidence of methamphetamine abuse using data for outpatient rehabilitants in Cape Town. Our estimated incidence of methamphetamine abuse, evaluated using the initiation function $\lambda S$, is observed to be decreasing sharply from the first half of the year 2000 down until the second half of the year 2002. It then suddenly increases from about 9 % in the year 2003 reaching an estimated incidence of 13 % in the second half of 2008, after which it steadily decreases until the first half of the year 2013

**Fig. 6:**
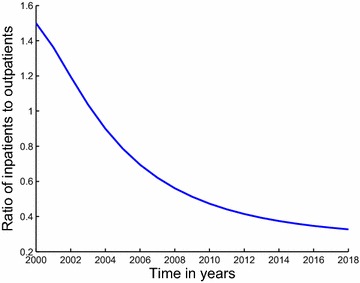
Estimation of the proportions of inpatient rehabilitants relative to outpatient rehabilitants in Cape Town projected over 5 more years. It demonstrates the changes of the ratio of inpatient rehabilitants to outpatient rehabilitants over the modelling time and projects these changes over a period of 5 years. The graph shows a steady decrease in the proportion inpatient rehabilitants coupled with an increase in the proportion of outpatient rehabilitants. The reason for the decrease is similar to the one used to for Figs. 2 and 3

The description of the model formulation and the model diagram lead to the following set of nonlinear ordinary differential equations together with the initial conditions (Figs. 5, 6):

2 $$\begin{aligned} \frac{dS}{dt}&\,=\,&\Pi -\lambda S-\mu S, \end{aligned}$$

3 $$\begin{aligned} \frac{dU}{dt}&\,=\,\lambda S +(1-\varepsilon )\rho _2 T_i \hfill \\ & \quad +(1-\varepsilon )\rho _1 T_0-(\mu +\sigma )U,\end{aligned}$$

4 $$\begin{aligned} \frac{dT_0}{dt}&\,=\,(1-p)\sigma U+\gamma _2 T_i\hfill \\ & \quad -(\mu +\gamma _1+(1-\varepsilon )\rho _1+\delta _0)T_0,\end{aligned}$$

5 $$\begin{aligned} \frac{dT_i}{dt}&\,=\, p\sigma U+\gamma _1T_0\hfill \\ & \quad -(\mu +\gamma _2+(1-\varepsilon )\rho _2+\delta _1)T_i, \end{aligned}$$

$$\begin{aligned} S(0)&\,=S_0>0,~U(0)\,\hfill \\ & =\,U_0\ge 0,~T_0(0)\,=\,T_{00}\ge 0,~T_i(0)\,=\,T_{i0}\ge 0. \end{aligned}$$

### Model analysis

#### Model properties

#### Positivity of solutions

**Fig. 7:**
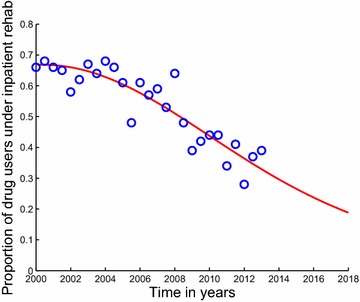
Model system Eqs. (2)–(5) fitted to data for individuals under inpatient rehabilitation in Cape Town and projected for 5 more years. The *blue circles* indicate the actual data and the *solid red line* indicates the model fit to the data. The percentages are not of all users, but of those in rehabilitation. The proportion of patients admitted under inpatient rehabilitation facilities in Cape Town is expected to be decreasing for the next five years, see also Fig. 6. This projected decrease in the proportion of inpatient rehabilitants can be an attribute of higher costs usually associated with inpatient rehabilitation programs and as a result, few individuals will afford receiving treatment at those facilities. As can be seen from the incidence curves related to data on inpatient rehabilitants, that inpatient rehabilitation programs have an increased potential of reducing methamphetamine incidence, there are fears that this decrease in the proportion of inpatient rehabilitants will to a greater extend result to higher prevalence rates of use observed for a long period of time

**Fig. 8:**
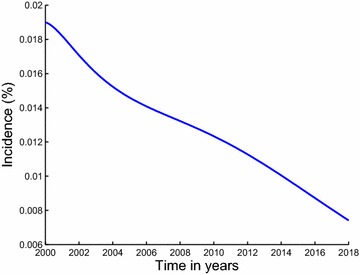
Estimated incidence of methamphetamine abuse using data for inpatient rehabilitants in Cape Town and projected for 5 more years. We also note the decreasing trend for incidence of methamphetamine abuse. Our estimated incidence will have decreased to below $7\,\%$ by the year 2018

We now consider the positivity of model system Eqs. (2)–(5). We prove that all the state variables remain non-negative and the solutions of model system Eqs. (2)–(5) with positive initial conditions will remain positive for all $t > 0$. We thus state the following theorem (Figs. 7, 8).

##### **Theorem 1**

*Given that the initial conditions of model system Eqs.* (2)–(5)* are*$S(0)>0$, $U(0)>0$, $T_0(0)>0$* and*$T_i(0)>0$.* There exists*$(S(t),U(t),T_0(t),T_i(t)): (0,\infty ) \rightarrow (0,\infty )$* which solve the model system Eqs.* (2)–(5).

##### *Proof*

Assume that

$$\begin{aligned} \hat{t}\,=\,\sup \{t>0:~S>0,~U>0,~T_0>0,~T_i>0\}\in [0,t]. \end{aligned}$$

Thus $\hat{t}>0$, and it follows from the first equation of model system Eqs. (2)–(5) that

$$\begin{aligned} & S(\hat{t})\exp \left( \mu \hat{t} + \displaystyle \int _0^{\hat{t}}{\lambda (s)\,ds}\right) \hfill \\ &-S(0)\ge \displaystyle \int _0^{\hat{t}}{\Pi \exp \left( \mu \hat{t} + \displaystyle \int _0^{\hat{t}}{\lambda (\nu )\,d\nu }\right) d\hat{t}}, \end{aligned}$$

giving

$$\begin{aligned} &S(\hat{t})\ge S(0)\exp \left[ -\left( \mu \hat{t} + \displaystyle \int _0^{\hat{t}}{\lambda (s)\,ds}\right) \right] \\ & +\exp \left[ -\left( \mu \hat{t} + \displaystyle \int _0^{\hat{t}}{\lambda (s)\,ds}\right) \right] \left[ \displaystyle \int _0^{\hat{t}}{\Pi \exp \left( \mu \hat{t} + \displaystyle \int _0^{\hat{t}}{\lambda (\nu )\,d\nu }\right) d\hat{t}}\right] >0. \end{aligned}$$

From the second equation of model system Eqs. (2)–(5), we obtain

$$\begin{aligned}&\frac{dU}{dt}\ge -(\mu +\sigma )U,\\&\Rightarrow U(\hat{t})\,=\,U_0 e^{-(\mu +\sigma )\hat{t}}>0. \end{aligned}$$

Similarly it can also be shown that $T_0(t)>0$ and $T_i(t)>0$ for all $t>0$, and this completes the proof. $\square$

#### Invariant region

##### **Theorem 2**

*The feasible region *$\mathcal {G}$* defined by*

$$\begin{aligned} \mathcal {G}\,=\,\left\{ (S,U,T_0,T_i)\in \mathbb {R}^4_+~|~0\le N\le \frac{\Pi }{\mu }\right\}, \end{aligned}$$

*with initial conditions*$S_0\ge 0$, $U_0\ge 0$, $T_{00}\ge 0$* and*$T_{i0}\ge 0$* is positively invariant and attracting with respect to model system Eqs.* (2)–(5) for all $t>0$.

##### *Proof*

Summing up Eqs. (2)–(5), we obtain that the total population satisfies the differential equation

6 $$\begin{aligned} \frac{dN}{dt}\le \Pi -\mu N. \end{aligned}$$

Applying a theorem by Birkhoff and Rota [22], on differential inequalities, we have

$$\begin{aligned} 0\,\le\,N(t)\le \frac{\Pi }{\mu }+N(0)e^{-\mu t}, \end{aligned}$$

where *N*(0) represents the value of Eqs. (2)–(5) evaluated at the initial values of the respective variables. Taking the limit as $t\rightarrow \infty$, we have that $0\le N\le \dfrac{\Pi }{\mu }$. Thus, the state variables remain biologically meaningful in the set

$$\begin{aligned} \mathcal {G}\,=\,\left\{ (S,U,T_0,T_i)\in \mathbb {R}^4_+| 0\le N\le \dfrac{\Pi }{\mu }\right\} , \end{aligned}$$

for all positive initial conditions in $\mathbb {R}^4_+$. Thus $\mathcal {G}$ is a positively invariant region and all solutions of Eqs. (2)–(5), with $(S_0,U_0,T_{00},T_{i0})\in \mathbb {R}^4_+$ remain in $\mathcal {G}$ for all $t>0$. Therefore the $\omega -\text{ limit }$ set of solutions of Eqs. (2)–(5) in $\mathcal {G}$ are contained in $\mathcal {G}$. The uniqueness, existence and continuity results hold for Eqs. (2)–(5). The system is thus mathematically and epidemiologically well-posed, see also [4]. Our analysis will be based on the dynamics of the solutions generated in $\mathcal {G}$. $\square$

### Methamphetamine-free equilibrium and the abuse reproduction number

In this section, we carry out stability analysis of the methamphetamine-free equilibrium. The model has a methamphetamine-free equilibrium given by

$$\begin{aligned} \mathcal {G}_0\,=\,(S_0,U_0,T_{00},T_{i0})\,=\,\left( \frac{\Pi }{\mu },0,0,0\right) , \end{aligned}$$

a scenario depicting a methamphetamine-free state in the community or society. The abuse reproduction number of the model, denoted $\mathcal {R}_a$, is the average number of secondary cases generated by one drug user during his/her duration of drug use in a population of completely potential drug users. The determination of $\mathcal {R}_a$ through the next generation matrix [23] method has been explored in many papers (see for instance [24–29]). Using the same method we have

$$\begin{aligned} \mathcal {R}_a\,=\,\mathcal {R}_U +\mathcal {R}_{T_0}+\mathcal {R}_{T_i} \end{aligned}$$

where

$$\begin{aligned} \mathcal {R}_U&\,=\,&\left( \frac{\beta }{g_1}\right) \left( \frac{1-\Psi _1}{1-(\Psi _1 +\Psi _2 +\Psi _3 +\Psi _4 +\Psi _5)}\right) ,\\ \mathcal {R}_{T_0}&\,=\,&\left( \frac{p\sigma }{g_1}\right) \left( \frac{\beta \eta }{g_2}\right) \left( \frac{\gamma _2}{g_3}\right) \left( \frac{1}{1-(\Psi _1 +\Psi _2 +\Psi _3 +\Psi _4 +\Psi _5)}\right) ,\\ \mathcal {R}_{T_i}&\,=\,&\left( \frac{(1-p)\sigma }{g_1}\right) \left( \frac{\beta \eta }{g_2}\right) \left( \frac{1}{1-(\Psi _1 +\Psi _2 +\Psi _3 +\Psi _4 +\Psi _5)}\right) ,\\ \text{ with }~g_1&\,=\,&\mu +\sigma ,~ g_2\,=\,\mu +\gamma _1 +(1-\varepsilon )\rho _1 +\delta _0,\\ g_3&\,=\,&\mu +\gamma _2 +(1-\varepsilon )\rho _2 +\delta _1, \\ \Psi _1&\,=\,&\frac{\gamma _1\gamma _2}{g_2g_3},\\ \Psi _2&\,=\,&\frac{(1-p)(1-\varepsilon )\rho _1\sigma }{g_1g_2},\\ \Psi _3&\,=\,&\frac{p(1-\varepsilon )\rho _2\sigma }{g_1g_3}, \\ \Psi _4&\,=\,&\frac{p(1-\varepsilon )\gamma _2\rho _1\sigma }{g_1g_2g_3}~\text{ and } \\ \Psi _5&\,=\,&\frac{(1-p)(1-\varepsilon )\gamma _1\rho _2\sigma }{g_1g_2g_3}. \end{aligned}$$

Here, $\mathcal {R}_a$ is the sum of three sub-reproduction numbers representing the contributions of individuals in compartments *U*, $T_0$ and $T_i$ respectively. The term $\Psi _1$ is the proportion of individuals who move back and forth between compartments $T_0$ and $T_i$, $\Psi _2$ is the proportion of individuals who move back and forth between compartments $T_0$ and *U* and $\Psi _3$ is the proportion of individuals who move back and forth between compartments $T_i$ and *U*. Interestingly, $\Psi _4$ is the proportion of those individuals who were once inpatient rehabilitants and have relapsed after joining outpatient rehabilitation and lastly, $\Psi _5$ is the proportion of those individuals who were once outpatient rehabilitants and have relapsed after joining inpatient rehabilitation facilities. The terms $\Psi _4$ and $\Psi _5$ play an essential role in evaluating the effectiveness of inpatient and outpatient rehabilitation programs.

The following result follows from [23].

#### **Theorem 3**

*The methamphetamine-free equilibrium point *$\mathcal {G}_0$* of model Eqs. *(2)–(5) is locally asymptotically stable for $\mathcal {R}_a\le 1$* and unstable otherwise.*

### Global stability of the methamphetamine-free steady state

We shall now prove the global stability of the methamphetamine-free equilibrium point $\mathcal {G}_0$ whenever the reproduction number is less than unity.

#### **Theorem 4**

*The methamphetamine-free equilibrium point*$\mathcal {G}_0$* of model Eqs.* (2)–(5) is globally* asymptotically stable for *$\mathcal {R}_a\le 1$* and unstable if*$\mathcal {R}_a>1$.

#### *Proof*

Let $V(U,T_0,T_i)\,=\,aU+bT_0+cT_i$ be a candidate Lyapunov function for some non-negative constants *a*, *b* and *c*. The time derivative of *V* is given by

$$\begin{aligned} \dot{V}&\,=\,a\dot{U}+b\dot{T_0}+c\dot{T_i}\\ &\quad \le {} a[\beta (U+\eta T_0) +(1-\varepsilon )\rho _2T_i +(1-\varepsilon )\rho _1T_0-g_1U]+b[(1-p)\sigma U+\gamma _2T_i-g_2T_0]\\&\quad+c[p\sigma U+\gamma _1T_0-g_3T_i]\left( \text{ since }~0\le \frac{S}{N}\le 1\right) \\& = [a(\beta -g_1)+b(1-p)\sigma +cp\sigma ]U+[a(\beta \eta +(1-\varepsilon )\rho _1)-bg_2+c\gamma _1]T_0\\&\quad+[a(1-\varepsilon )\rho _2+b\gamma _2-cg_3]T_i. \end{aligned}$$

We now evaluate the coefficients of the suitable Lyapunov function such that the coefficients of $T_0$ and $T_i$ are equal to zero. We thus obtain

$$\begin{aligned} a&= g_2g_3-\gamma _1\gamma _2,\\ b &=(\beta \eta +(1-\varepsilon )\rho _1)g_3+(1-\varepsilon )\gamma _1\rho _2\quad \text{ and }\\ c&=(\beta \eta +(1-\varepsilon )\rho _1)\gamma _2+(1-\varepsilon )g_2\rho _2. \end{aligned}$$

Using these coefficients, the time derivative of the Lyapunov function can be expressed as

$$\begin{aligned} \frac{dV}{dt}\,\le\, g_1g_2g_3\left[ \left( 1-\left( \Psi _1 +\Psi _2 +\Psi _3 +\Psi _4 +\Psi _5\right) \right) \left( \mathcal {R}_a-1\right) \right] U. \end{aligned}$$

We can deduce that $\dfrac{dV}{dt}\le 0$ when $\mathcal {R}_a\le 1$ with equality if $\mathcal {R}_a\,=\,1$. Furthermore, $\dfrac{dV}{dt}\,=\,0$ if and only if $U\,=\,T_0\,=\,T_i\,=\,0$. Therefore, the largest compact invariant set in $(S,U,T_0,T_i)\in \mathcal {G}$ such that $\dfrac{dV}{dt}\,=\,0$ when $\mathcal {R}_a\le 1$ is the singleton $\mathcal {G}_0$. By Lasalle invariance principle [30], this implies that $\mathcal {G}_0$ is globally stable in $\mathcal {G}$ if $\mathcal {R}_a\le 1$. We observe that the Jacobian matrix evaluated at $\mathcal {G}_0$ has a positive eigenvalue whenever $\mathcal {R}_a>1$. Therefore the methamphetamine-free equilibrium is unstable if $\mathcal {R}_a>1$. This completes the proof. $\square$

### The methamphetamine persistent equilibrium point

In this section we determine the methamphetamine persistent equilibrium point by first setting the left hand side of model Eqs. (2)–(5) to zero. Some easy to follow algebraic manipulations give the methamphetamine-persistent equilibrium $\mathcal {G}^*\,=\,(S^*,U^*,T^*_0,T^*_i)$ where

7 $$\begin{aligned}&S^*\,=\,\frac{A\Pi }{A\mu +\Theta (\mathcal {R}_a-1)},\quad U^*\,=\,\frac{\Pi g_2g_3(1-\Psi _1)(\mathcal {R}_a-1)}{(p\sigma \gamma _2 +g_3(1-p)\sigma )(A\mu +\Theta (\mathcal {R}_a-1))},\nonumber \\&T^*_0\,=\,\frac{\Pi (\mathcal {R}_a-1)}{A\mu +\Theta (\mathcal {R}_a-1)}, \quad T^*_i\,=\,\frac{\Pi (\mathcal {R}_a-1)[p\sigma g_2g_3(1-\Psi _1)+\gamma _1(p\sigma \gamma _2 +g_3(1-p)\sigma )]}{g_3(p\sigma \gamma _2 +g_3(1-p)\sigma )(A\mu +\Theta (\mathcal {R}_a-1))}. \end{aligned}$$

We thus have the following result:

#### **Theorem 5**

*Model system Eqs*. (2,3,4)–(5)* has a unique methamphetamine-persistent equilibrium whenever*$\mathcal {R}_a>1$.

The results on persistence of the model are given in [31]. For more information about persistence of dynamical systems, we refer the reader to [32–36].

### Global stability of the methamphetamine-persistent steady state

In this section, we prove the global stability of the methamphetamine-persistent steady state.

#### **Theorem 6**

*Assume that *$\gamma _1\,=\,\theta \gamma _2$* where*$\theta \,=\,\dfrac{p\rho _1}{(1-p)\rho _2}$.* If *$\mathcal {R}_a>1$,* then the unique methamphetamine-persistent equilibrium*$\mathcal {G}^*$* of model system Eqs.* (2)–(5)* is globally asymptotically stable in the interior of *$\mathcal {G}$.

#### *Proof*

The global asymptotic stability of the methamphetamine-persistent steady state is proved by constructing a global Lyapunov function following [37]. We propose the following Lyapunov function obtained from a suitable combination of common quadratic and Volterra-type functions. Define $\mathcal {V}:~\{(S,U,T_0,T_i)\in \mathcal {G}:~S,U,T_0,T_i>0\}\rightarrow \mathbb {R}$ by

$$\begin{aligned} \mathcal {V}&\,=\,\frac{(S-S^*)^2}{2S^*}+\left( U-U^*-U^*\ln \frac{U}{U^*}\right) +\frac{(1-\varepsilon )\rho _1}{(1-p)\sigma }\frac{T^*_0}{U^*}\left( T_0-T_0^*-T_0^*\ln \frac{T_0}{T^*_0}\right) \\&\quad +\frac{(1-\varepsilon )\rho _2}{p\sigma }\frac{T^*_i}{U^*}\left( T_i-T_i^*-T_i^*\ln \frac{T_i}{T^*_i}\right) . \end{aligned}$$

This function is defined, continuous and positive definite for all $S,U,T_0,T_i>0$. We also observe that $\mathcal {V}(S,U,T_0,T_i)\,=\,0$ at the steady state $\mathcal {G}^*$. Since $\mathcal {G}^*\,=\,(S^*,U^*,T^*_0,T^*_i)$ is an endemic steady state point of model system Eqs. (2)–(5), we have

8 $$\begin{aligned} \Pi&\,=\,\lambda ^*S^*+\mu S^*, \nonumber \\ g_1&\,=\,\lambda ^*\frac{S^*}{U^*}+(1-\varepsilon )\rho _2\frac{T^*_i}{U^*}+(1-\varepsilon )\rho _1\frac{T^*_0}{U^*},\\ g_2&\,=\,(1-p)\sigma \frac{U^*}{T^*_0}+\gamma _2\frac{T^*_i}{T^*_0}, \nonumber \\ g_3&\,=\,p\sigma \frac{U^*}{T^*_i}+\gamma _1\frac{T^*_0}{T^*_i}.\nonumber \end{aligned}$$

Computing the time derivative of $\mathcal {V}(S,U,T_0,T_i)$ along the solution of model system Eqs. (2)–(5), we obtain

9 $$\begin{aligned} \dot{\mathcal {V}}&\,=\,\left( \frac{S-S^*}{S^*}\right) \dot{S}+\left( \frac{U-U^*}{U}\right) \dot{U} \\ & \quad+\frac{(1-\varepsilon )\rho _1}{(1-p)\sigma }\frac{T^*_0}{U^*}\left( \frac{T_0-T^*_0}{T_0}\right) \dot{T_0}\nonumber \\&\quad+\frac{(1-\varepsilon )\rho _2}{p\sigma }\frac{T^*_i}{U^*}\left( \frac{T_i-T^*_i}{T_i}\right) \dot{T_i}. \end{aligned}$$

Using model system Eqs. (2)–(5) and (8), it can be easily shown that

10 $$\begin{aligned} \left( \frac{S-S^*}{S^*}\right) \dot{S}&\,=\,\left( \frac{S-S^*}{S^*}\right) (\Pi -\lambda S-\mu S)\nonumber \\&\,=\,-\beta U\frac{(S-S^*)^2}{S^*}\\ &\quad -\beta \eta T_0\frac{(S-S^*)^2}{S^*}-\mu \frac{(S-S^*)^2}{S^*}-\beta (S-S^*)(U-U^*)\nonumber \\& \quad -\beta \eta (S-S^*)(T_0-T^*_0). \end{aligned}$$

Similarly, using model system Eqs. (2)–(5) and (8), we can also show that

11 $$\begin{aligned} \left( \frac{U-U^*}{U^*}\right) \dot{U}&\,=\,(U-U^*) \\ & \quad \times\left[ \beta (S-S^*)+\beta \eta \left( \frac{T_0S}{U}-\frac{T^*_0S^*}{U^*}\right) +(1-\varepsilon )\rho _1\left( \frac{T_0}{U}-\frac{T^*_0}{U^*}\right) \right] \nonumber \\& \quad +(U-U^*)\left[ (1-\varepsilon )\rho _2\left( \frac{T_i}{U}-\frac{T^*_i}{U^*}\right) \right] . \end{aligned}$$

We also have

12 $$\begin{aligned} \frac{(1-\varepsilon )\rho _1}{(1-p)\sigma }\frac{T^*_0}{U^*}\left( \frac{T_0-T^*_0}{T_0}\right) \dot{T_0}&\,=\,\frac{T^*_0}{U^*}(T_0-T^*_0)\left[ (1-\varepsilon )\rho _1\left( \frac{U}{T_0}-\frac{U^*}{T^*_0}\right) \right] \nonumber \\&\quad +\frac{T^*_0}{U^*}(T_0-T^*_0)\left[ \frac{(1-\varepsilon )\rho _1}{(1-p)\sigma }\gamma _2\left( \frac{T_i}{T_0}-\frac{T^*_i}{T^*_0}\right) \right] . \end{aligned}$$

and

13 $$\begin{aligned} \frac{(1-\varepsilon )\rho _2}{p\sigma }\frac{T^*_i}{U^*}\left( \frac{T_i-T^*_i}{T_i}\right) \dot{T_i}&\,=\,\frac{T^*_i}{U^*}(T_i-T^*_i)\left[ (1-\varepsilon )\rho _2\left( \frac{U}{T_i}-\frac{U^*}{T^*_i}\right) \right] \nonumber \\&\quad +\frac{T^*_i}{U^*}(T_i-T^*_i)\left[ \frac{(1-\varepsilon )\rho _2}{p\sigma }\gamma _1\left( \frac{T_0}{T_i}-\frac{T^*_0}{T^*_i}\right) \right] . \end{aligned}$$

Adding expressions (10), (11), (12) and (13) gives

14 $$\begin{aligned} \dot{\mathcal {V}}&=\left( \frac{S-S^*}{S^*}\right) \dot{S}+\left( \frac{U-U^*}{U}\right) \dot{U}+\frac{(1-\varepsilon )\rho _1}{(1-p)\sigma }\frac{T^*_0}{U^*}\left( \frac{T_0-T^*_0}{T_0}\right) \dot{T_0}\nonumber \\&\quad+\frac{(1-\varepsilon )\rho _2}{p\sigma }\frac{T^*_i}{U^*}\left( \frac{T_i-T^*_i}{T_i}\right) \dot{T_i}\nonumber \\&=A_0-\beta \eta (S-S^*)(T_0-T^*_0)+\beta \eta (U-U^*)\left( \frac{T_0S}{U}-\frac{T^*_0S^*}{U^*}\right) \nonumber \\&+(1-\varepsilon )\rho _1(U-U^*)\left( \frac{T_0}{U}-\frac{T^*_0}{U^*}\right) \nonumber \\&\quad +\frac{T^*_0}{U^*}(1-\varepsilon )\rho _1(T_0-T^*_0)\left( \frac{U}{T_0}-\frac{U^*}{T^*_0}\right) \nonumber \\&\quad +(1-\varepsilon )\rho _2(U-U^*)\left( \frac{T_i}{U}-\frac{T^*_i}{U^*}\right) \nonumber \\&\quad +(1-\varepsilon )\rho _2\frac{T^*_i}{U^*}(T_i-T^*_i)\left( \frac{U}{T_i}-\frac{U^*}{T^*_i}\right) \nonumber \\&\quad +\frac{(1-\varepsilon )\rho _1\gamma _2}{(1-p)\sigma }\frac{T^*_0}{U^*}(T_0-T^*_0)\left( \frac{T_i}{T_0}-\frac{T^*_i}{T^*_0}\right) \nonumber \\\nonumber&\quad +\frac{(1-\varepsilon )\rho _2\gamma _1}{p\sigma }\frac{T^*_i}{U^*}(T_i-T^*_i)\left( \frac{T_0}{T_i}-\frac{T^*_0}{T^*_i}\right) \nonumber \\&=A_0+\beta \eta \left[ ST^*_0+S^*T_0-T^*_0S^*\frac{U}{U^*}-T_0S\frac{U^*}{U}\right] \nonumber \\&\quad +(1-\varepsilon )\rho _1T^*_0\left[ \frac{T_0}{T^*_0}-\frac{U}{U^*}-\frac{T_0}{T^*_0}\frac{U^*}{U}+1\right] \nonumber \\&\quad +(1-\varepsilon )\rho _1T^*_0\left[ \frac{U}{U^*}-\frac{T_0}{T^*_0}-\frac{T^*_0}{T_0}\frac{U}{U^*}+1\right] \nonumber \\&\quad +(1-\varepsilon )\rho _2T^*_i\left[ \frac{T_i}{T^*_i}-\frac{U}{U^*}-\frac{T_i}{T^*_i}\frac{U^*}{U}+1\right] \nonumber \\&\quad +(1-\varepsilon )\rho _2T^*_i\left[ \frac{U}{U^*}-\frac{T_i}{T^*_i}-\frac{T^*_i}{T_i}\frac{U}{U^*}+1\right] \nonumber \\&\quad +\frac{(1-\varepsilon )\rho _1\gamma _2}{(1-p)\sigma }\frac{T^*_0T^*_i}{U^*}\left[ \frac{T_i}{T^*_i}-\frac{T_0}{T^*_0}-\frac{T^*_0}{T_0}\frac{T_i}{T^*_i}+1\right] \nonumber \\&\quad +\frac{(1-\varepsilon )\rho _2\gamma _1}{p\sigma }\frac{T^*_0T^*_i}{U^*}\left[ \frac{T_0}{T^*_0}-\frac{T_i}{T^*_i}-\frac{T_0}{T^*_0}\frac{T^*_i}{T_i}+1\right] \nonumber \\&=A_0+\beta \eta S^*T_0\left[ 1+\frac{S}{S^*}\frac{T^*_0}{T_0}-\frac{T^*_0}{T_0}\frac{U}{U^*}-\frac{S}{S^*}\frac{U^*}{U}\right] \nonumber \\&\quad +(1-\varepsilon )\rho _1T^*_0\left[ 2-\frac{U^*}{U}\frac{T_0}{T^*_0}-\frac{U}{U^*}\frac{T^*_0}{T_0}\right] \nonumber \\&\quad +(1-\varepsilon )\rho _2T^*_i\left[ 2-\frac{U^*}{U}\frac{T_i}{T^*_i}-\frac{U}{U^*}\frac{T^*_i}{T_i}\right] \nonumber \\&\quad +\frac{(1-\varepsilon )\rho _2\gamma _1}{p\sigma }\frac{T^*_0T^*_i}{U^*}\left[ 2-\frac{T^*_0}{T_0}\frac{T_i}{T^*_i}-\frac{T_0}{T^*_0}\frac{T^*_i}{T_i}\right] ~(\text{ since }~\gamma _1\,=\,\theta \gamma _2)\nonumber \\&=A_0-\beta \eta S^*T_0\left[ 3-\left( \frac{S}{S^*}\frac{T^*_0}{T_0}+\frac{T_0}{T^*_0}\frac{U^*}{U}+\frac{S^*}{S}\frac{U}{U^*}\right) \right] \nonumber \\&\quad -\beta \eta S^*T_0\left[ \sqrt{\frac{T^*_0}{T_0}\frac{U}{U^*}}-\sqrt{\frac{T_0}{T^*_0}\frac{U^*}{U}}\right] ^2\nonumber \\&\quad -\beta \eta S^*T_0\left[ \sqrt{\frac{S}{S^*}\frac{U^*}{U}}-\sqrt{\frac{S^*}{S}\frac{U}{U^*}}\right] ^2-(1-\varepsilon )\rho _1T^*_0\left[ \sqrt{\frac{T_0}{T^*_0}\frac{U^*}{U}}-\sqrt{\frac{T^*_0}{T_0}\frac{U}{U^*}}\right] ^2\nonumber \\&\quad -(1-\varepsilon )\rho _2T^*_i\left[ \sqrt{\frac{T_i}{T^*_i}\frac{U^*}{U}}-\sqrt{\frac{T^*_i}{T_i}\frac{U}{U^*}}\right] ^2-\frac{(1-\varepsilon )\rho _2\gamma _1}{p\sigma }\frac{T^*_0T^*_i}{U^*}\left[ \sqrt{\frac{T^*_0}{T_0}\frac{T_i}{T^*_i}}-\sqrt{\frac{T_0}{T^*_0}\frac{T^*_i}{T_i}}\right] ^2, \end{aligned}$$

where

$$\begin{aligned} A_0\,=\,-\beta U\frac{(S-S^*)^2}{S^*}-\beta \eta T_0\frac{(S-S^*)^2}{S^*}-\mu \frac{(S-S^*)^2}{S^*}\le 0. \end{aligned}$$

**Fig. 9:**
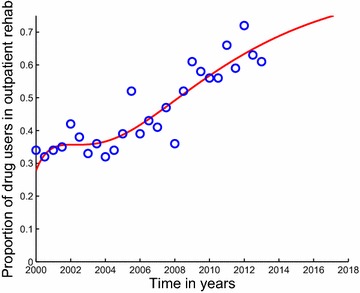
Model system Eqs. (2)–(5) fitted to data for individuals under outpatient rehabilitation in Cape Town and projected for five more years. The *blue circles* indicate the actual data and the *solid red line* indicates the model fit to the data. The proportion of patients admitted under outpatient rehabilitation facilities in Cape Town is likely to continue increasing for the next five years, see also Fig. 6. The percentages are not of all users, but of those in rehabilitation

**Fig. 10:**
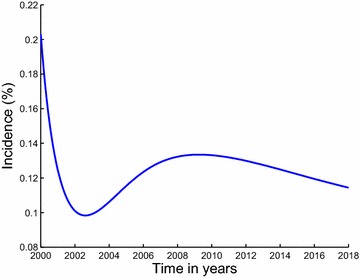
Estimated incidence of methamphetamine abuse using data for outpatient rehabilitants in Cape Town and projected for five more years. The estimated incidence of methamphetamine abuse is expected to gradually decrease for the next five years down to below $11.5\,\%$ by the year 2018

We observe from expression (14) that (Figs. 9, 10)

$$\begin{aligned} \left( \dfrac{S}{S^*}\dfrac{T^*_0}{T_0}+\dfrac{T_0}{T^*_0}\dfrac{U^*}{U}+\dfrac{S^*}{S}\dfrac{U}{U^*}\right) \,=\, 3 \end{aligned}$$

only if $$S\,=\,S^*,~U\,=\,U^*\quad \text{ and }\quad 
T_0\,=\,T^*_0$$. Thus we can deduce that, $$\dot{\mathcal {V}(S,U,T_0,T_i)}$$ is negative definite if

$$\begin{aligned} \left( \dfrac{S}{S^*}\dfrac{T^*_0}{T_0}+\dfrac{T_0}{T^*_0}\dfrac{U^*}{U}+\dfrac{S^*}{S}\dfrac{U}{U^*}\right) < 3 \end{aligned}$$

and $$\dot{\mathcal {V}(S,U,T_0,T_i)}\,=\,0$$ only if $$S\,=\,S^*,~U\,=\,U^*,~T_0\,=\,T^*_0 \quad \text{ and } \quad T_i\,=\,T^*_i$$. Therefore, the largest compact invariant set in $$\{(S,U,T_0,T_i)\in \mathcal {G}:$$$$\dot{\mathcal {V}(S,U,T_0,T_i)}\,=\,0\}$$ is the singleton $$\{{\mathbf {G}}^{*}\}$$. By LaSalle’s Invariance Principle [30], we conclude that $${\mathbf {G}}^*$$ is globally asymptotically stable in the interior of $$\mathcal {G}$$. This completes the proof. $$\square$$

## Results and discussion

### Numerical simulations

#### Data

In this section we present an application of our model through fitting the model data on rehabilitation from the Medical Research Council’s (MRC’s), South African Community Epidemiology Network on Drug Use (SACENDU) project [40]. We fit the model system Eqs. (2)–(5) to data for individuals under inpatient and outpatient rehabilitation in Cape Town. We use data in Table 1 which was collected by the SACENDU for individuals who were admitted on inpatient and outpatient forms of rehabilitation in Cape Town.

While inpatient and outpatient rehabilitation programs may have similar components, they can have very different long-term outcomes and success rates. Statistics on success rate of substance abuse rehabilitation programs vary widely [39] and depend on a variety of factors, such as, the extent and nature of the patient’s problems, the appropriateness of treatment and related services used to address those problems, and the quality of interaction between the patient and his or her treatment providers.

**Table 1:** Treatment type received for the period 1999a to 2013a (%)

Year	1999a	1999b	2000a	2000b	2001a	2001b	2002a	2002b
Inpatient (%)	69	66	66	68	66	65	58	62
Outpatient (%)	27	32	34	32	34	35	42	38

Year	2003a	2003b	2004a	2004b	2005a	2005b	2006a	2006b
Inpatient (%)	67	64	68	66	61	48	61	57
Outpatient (%)	33	36	32	34	39	52	39	43

Year	2007a	2007b	2008a	2008b	2009a	2009b	2010a	2010b
Inpatient (%)	59	53	64	48	39	42	44	44
Outpatient (%)	41	47	36	52	61	58	56	56

Year	2011a	2011b	2012a	2012b	2013a			
Inpatient (%)	34	41	28	37	39			
Outpatient (%)	66	59	72	63	61			

Letter ‘a’ represents the first 6 months of the year and ‘b’ represents the last 6 months of the year

#### Parameter estimation

We make use of Matlab programming language to estimate model parameters used in our numerical simulations and to analyze existing trends on inpatient and outpatient forms of rehabilitation. The model parameters that we use for numerical simulations are in Table 2. We make use of curve fitting, which is a process that allows us to quantitatively estimate the trend of the outcomes. The curve fitting process fits equations of approximating curves to the raw field data. Nevertheless, for a given set of data, the fitting curves of a given type are generally not unique. Thus, a curve with a minimal deviation from all data points is desired. This best-fitting curve can be obtained by the method of least squares. The least squares curve fit routine (lsqcurvefit) in Matlab with optimization is used to estimate the parameter values. Many parameters are known to lie within some intervals. During the estimation of parameter values, a Matlab code is used where unknown parameter values are given a lower and upper bound from which the set of parameter values that provide the best fit are obtained. The intervals used and a few parameters obtained from literature are given in Table 2.

**Table 2:** Parameter values and ranges obtained from data fitting

	Description	Range	Value	Source
$\beta$	The effective contact rate between users and susceptibles	0.10–0.21	$0.105\, \mathrm{year}^{-1}$	[15]
$\eta$	The relative ability for outpatients to initiate new users	0–0.0099	$0.009612\, \mathrm{year}^{-1}$	Estimated
*p*	Proportion of users recruited into inpatient rehab	0–1	$0.352~\mathrm{year}^{-1}$	Estimated
$\varepsilon$	The effectiveness of rehab	0–1	$0.6080\, \mathrm{year}^{-1}$	Estimated
$\sigma$	The rate at which users are recruited into rehab	0–0.05024	$0.02827\, \mathrm{year}^{-1}$	Estimated
$\delta _0$	The rate of quitting abuse for outpatients	0.001–1	$0.01\, \mathrm{year}^{-1}$	Estimated
$\delta _1$	The rate of quitting abuse for inpatients	0.01–1	$0.3142~\mathrm{year}^{-1}$	Estimated
$\rho _1$	Relapse rate for outpatients	0–0.054	$0.0382\, \mathrm{year}^{-1}$	Estimated
$\rho _2$	Relapse rate for inpatients	0–0.0235	$0.0020\, \mathrm{year}^{-1}$	Estimated
$\gamma _1$	Transfer rate from outpatient rehab to inpatient rehab	0–0.06012	$0.02961\, \mathrm{year}^{-1}$	Estimated
$\gamma _2$	Transfer rate from inpatient rehab to outpatient rehab	0–0.008	$0.003\, \mathrm{year}^{-1}$	Estimated
$\Pi$	Recruitment rate into the susceptible population	0.028–0.080	$0.04\, \mathrm{year}^{-1}$	[15]
$\mu$	Natural death rate	0.019–0.021	$0.020\, \mathrm{year}^{-1}$	[41]

*MA* methamphetamine

## Conclusions

In this paper, we designed a model that incorporates inpatient and outpatient rehabilitation of methamphetamine addicts to study the dynamics of methamphetamine abuse in Cape Town of South Africa. The reproduction number was derived and qualitatively used to investigate the stability of equilibrium states and the prevalence of methamphetamine abuse. The methamphetamine-free equilibrium point is shown to be globally asymptotically stable whenever the reproduction number is less than unity. Thus, methamphetamine abuse can be eliminated if control strategies are put in place and efforts are directed toward reducing the threshold number to a value less than unity. With the aid of a Lyapunov function obtained from a suitable combination of common quadratic and Volterra-type functions, the methamphetamine-persistent steady state has been shown to be globally asymptotically stable whenever the reproduction number is greater than unity.

The least squares curve fit routine (lsqcurvefit) in Matlab with optimization has been used to fit the model to data on inpatient and outpatient rehabilitants with the objective of using the model parameters that give the best fit to obtain the incidence curve. We also used the parameter values that give the best fit to plot a graph of the ratio of inpatients to outpatients and as well predict future proportions of inpatient and outpatient rehabilitants. The results suggest that the proportion of patients admitted under inpatient rehabilitation facilities in Cape Town will continue to decrease for the next five years whereas that for outpatient rehabilitants will increase for the next five years. The estimated proportion of inpatient rehabilitants in specialist treatment centres of Cape Town was observed to be approximately $31\,\%$ by the year 2018. Our estimated incidence of methamphetamine abuse related to data on inpatient and outpatient rehabilitants was observed to be generally decreasing over the years. However, it was noted that the estimated incidence for methamphetamine abuse related to data on inpatient rehabilitants had a sharp decrease as compared to that of outpatient rehabilitants, suggesting that inpatient rehabilitation programs have an increased potential of positively changing the lives of many methamphetamine addicts. The projections carried out show that the estimated incidence for methamphetamine abuse related to data on inpatient rehabilitants will have decreased down to below $7\,\%$ by the year 2018 and that of outpatient rehabilitants will have decreased to below $11.5\,\%$ by the year 2018. The study presented here is not exhaustive, it can be extended to include stratification of the population according to levels of methamphetamine use. Structuring the population in such a way would give some other helpful insights in studying the dynamics of methamphetamine abuse. In addition, the model did not take into account contextual dynamics, such as drug supply chains or changes in interdiction. Incorporating these processes will undoubtedly facilitate in the understanding of methamphetamine dynamics. Also, since the study focuses specifically on methamphetamine abuse, the model dynamics may also be affected by the availability and use of other drugs.

## Abbreviations

SUD: Substance use disorder
DOSD: Department of Social Development
MRC: Medical Research Council
SACENDU: South African Community Epidemiology Network on Drug Use
MA: methamphetamine
